# Sudden cardiac death in young First Nations Australians in the Northern Territory, Australia: Potential implications for pre-participation screening

**DOI:** 10.1016/j.jsampl.2025.100100

**Published:** 2025-04-21

**Authors:** Sonali Pande, Viran DeSilva, Elizabeth Paratz, Marianne Tiemensma, Nadarajah Kangaharan

**Affiliations:** 1 Territory Sportsmedicine, Parap, Darwin, NT, 0800, Australia; 2 Faculty of Medicine, Dentistry & Health Sciences, University of Melbourne, 300 Grattan St Parkville VIC 3000, Australia; 3 Heart, Exercise & Research Trials Laboratory, St Vincent's Institute, 9 Princes St, Fitzroy, VIC, 3065, Australia; 4 Specialist Forensic Pathologist, Royal Darwin Hospital, 0810, Tiwi, NT, Australia; 5 College of Medicine and Public Health, Flinders University, Adelaide, Australia; 6 Cardiology Department, Royal Darwin Hospital, 0810, Tiwi, NT, Australia; 7 NT Cardiac, Royal Darwin Hospital, Darwin, 0810, Tiwi, NT, Australia; 8 Menzies School of Health Research, Royal Darwin Hospital, 0810, Tiwi, NT, Australia

**Keywords:** Sudden cardiac death, Cardiovascular disease, First Nations Australians, Screening

## Abstract

**Objective:**

To present data from coronial records on sudden cardiac death (SCD) cases seen in young, First Nations Australians in the Northern Territory of Australia, estimate its incidence, and propose potential pre-participation screening strategies.

**Design:**

Retrospective observational study.

**Methods:**

Coronial records of sudden cardiac death cases in First Nations Australians in the Northern Territory under 40 years of age occurring between 2019 and 2023 were reviewed to study the incidence, demographics, medical history, circumstances of death and causes of death with autopsy and toxicology analysis.

**Results:**

A total of 59 SCD cases in First Nations Australians under 40 years of age were recorded in the Northern Territory with an annual incidence of 19.8 cases per 100,000 persons. The mean ​± ​SD of age was 32.8 ​± ​6.14 years. There were 61 ​% male and 2/3 of SCD cases occurred in remote location. Coronary heart disease (n ​= ​36; 61 ​%) was the most common cause of death. In 3 cases, SCD was related to sports or exercise activity. Most common medical co-morbidities were cardiac (38.9 ​%), Diabetes mellitus (35.6 ​%), and rheumatic heart disease (20.3 ​%). Smoking (37.3 ​%) and alcohol abuse (32.2 ​%) were the most common risk factors.

**Conclusions:**

SCD is more common and coronary heart disease and rheumatic heart disease are the most common causes in First Nations Australians in the Northern Territory under the age of 40 years. Medical co-morbidities and risk factors are prevalent in this population. There is a need for First Nation Australians specific local guidelines for a comprehensive pre-participation Heart-Health assessment.

## 1 Introduction

Sudden cardiac death (SCD) refers to an unexpected fatality from cardiac causes. First Nations Australians face higher rates of cardiovascular diseases-related deaths at younger ages with rates of CHD prevalence, hospitalisations, deaths and burden of disease more than twice as compared with non- First Nations Australians [1]. SCD may result from a diverse number of aetiologies, affecting the structure and/or function of the heart [2]. Amongst First Nations individuals, rheumatic heart disease (RHD) remains near-endemic and premature coronary heart disease (CHD) is disproportionately high [3].

Cardiovascular disease (CVD) is a leading cause of death in Australia, accounting for 30 ​% of all deaths. In 2018, the burden of CVD among First Nations Australians was 2.4 times that of non-First Nations Australians [4]. They are twice as likely to die from CVD than non-First Nations Australians [5], with earlier age of onset and demonstrable inequalities in cardiac care [3,[6], [7], [8]].

First Nations Australians, represent 3.8 ​% of the total Australian population, with the highest proportion living in the Northern Territory (30.8 ​%) relative to its total population size [9]. The national gap in life expectancy between First Nations and non-First Nations Australians was 8.6 years for males and 7.8 years for females [10]. For First Nations Australians in the Northern Territory, the life expectancy at birth was 67 years and 70 years for males and females respectively [11].

It is anticipated that CVD may be more common amongst young First Nations Australians in the Northern Territory as an increasing number take part in competitive sports. The aim of the study was to present data from coronial records on SCD cases seen under 40 years of age, estimate its incidence in First Nations Australians in the Northern Territory and propose potential pre-participation screening strategies for consideration.

## 2 Methods

In a retrospective study, coronial records of SCD cases in First Nations Australians under 40 years of age in the Northern Territory between 2019 and 2023 were identified and reviewed. From the records available with the Royal Darwin Hospital Forensic Pathology Unit, information on baseline demographics, medical history, and circumstances of death including previous symptoms was obtained. The causes of death were obtained from the final autopsy report when performed. A consensus on the cause of death was reached in a meeting between the co-authors (NK, MT) attended by other co-authors (SP, VD). The annual incidence was estimated based on First Nations Australian population in Northern Territory in 2021.

The data is presented using descriptive statistics. Continuous variables like age and BMI are presented as mean ​± ​SD while categorical variables are presented as numbers (n) (%; percentage of sample).

Ethics approval was obtained from the Human Research Ethics Committee of NT (Northern Territory) Health and Menzies School of Health Research, and permission to use coronial data was granted by the NT Coroner (NT HREC Reference Number: 2023–4766, NT Health SSA: EFILE 2023/28848).

‘First Nations Australians’ are the Aboriginal and Torres Strait Islander people in Australia, self-identified in the Northern Territory Health's electronic records. They are also referred to as ‘Indigenous Australians’. ‘First Nations Australians’ is used across this article to respectfully address this population.

## 3 Results

A total of 59 SCD cases in First Nations Australians were recorded in the coronial population during the study period. Autopsy were performed in 44 cases (74.6 ​%) while in 15 cases autopsies were not required as the cause of death was clear following review of available background medical and circumstantial information by the forensic pathologist.

The estimated annual incidence of SCD in First Nations Australians was 19.8 per 100,000 persons.

The characteristics of the sample are presented in Table 1. The mean ​± ​SD of age was 32.8 ​± ​6.14 years with a range of 14–40 years. There were 61 ​% (36/59) male and 16.9 ​% (10/59) patients had undergone some type of cardiac intervention. Two thirds of SCD occurred in remote locations with 61 ​% (36/59) at home.

**Table 1:** Characteristics of SCD cases in First Nations Australians under 40 years in the Northern Territory (2019–2023).

	n ​= ​59, n (%)
Age, y: mean ​± ​SD	32.8 ​± ​6.14
M,F	36,23 (61 ​%, 39 ​%)
BMI, kg/m^2^: Mean ​± ​SD	26.1 ​± ​6.7 (n ​= ​44)
BMI >30 ​kg/m^2^	17 (28.8)
**Residential location**	
Regional	11 (18.6)
Remote	44 (74.6)
Unknown	4 (6.8)
**Comorbidities**	
Cardiac	23 (38.9)
Diabetes mellitus	21 (35.6)
Hyperlipidaemia	16 (27.1)
Renal disease	16 (27.1)
Hypertension	15 (25.4)
Rheumatic heart disease	12 (20.3)
**Risk factors**	
Smoking	22 (37.3)
Alcohol	19 (32.2)
Obesity	18 (30.5)
Substance abuse	18 (30.5)
**Pre-SCD symptoms**	
Chest pain	15 (25.4)
Shortness of breath	7 (11.9)
Seizures	5 (8.5)
Vomiting	2 (3.4)
**Co-existing cardiac diagnoses potentially contributing to SCD**	
Rheumatic valvular heart disease	5 (8.8)
Hypertensive heart disease	7 (11.9)
Left ventricular failure in RHD	5 (8.8)
Coronary heart disease	2 (3.4)
Cardiomegaly	2 (3.4)
Long QT syndrome	1 (1.7)
Myocarditis	1 (1.7)
Atrial fibrillation in RHD	1 (1.7)
**Previous cardiac procedures**	
Valve replacement	6 (10.1)
Coronary stenting	3 (5)
Pacemaker	1 (1.7)
**Circumstances of SCD**	
At home	36 (61)
On the street	7 (11.8)
During hospital attendance	7 (11.8)
Related to sports and exercise	3 (5)
At worksite	2 (3.3)
Following altercation	1 (1.7)
Not recorded	3 (5)

**Fig. 1:**
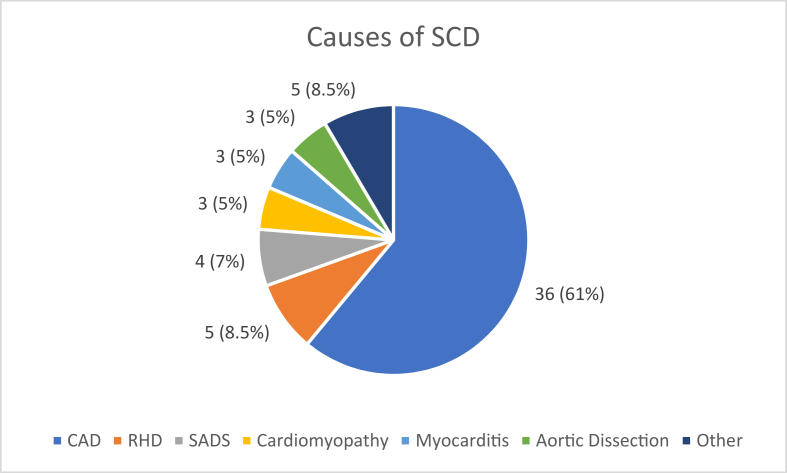
Primary causes of SCD in First Nations Australians under 40 years in the Northern Territory (2019–2023).

The primary causes of death noted in the sample are given in Fig. 1.

The 3 cases of SCD related to sports or exercise activity are summarized here.

### 3.1 Case 1

A 35-year-old male suffered seizures and was found unresponsive in a remote location, shortly after a cricket match in which he batted for a prolonged period of time. Cardiopulmonary resuscitation (CPR) was performed with the use of an Automated External Defibrillator (AED) but he could not be revived. A review of his records showed that he was obese, a smoker and had hypertension, hyperlipidaemia and had undergone cardiac stenting 2 years prior. The cause of death was recorded as ischaemic heart disease secondary to coronary artery disease. Autopsy revealed ischaemic cardiomyopathy with extensive myofibrosis of the posterior wall. A stent was noted in the LAD and there was 50–60 ​% stenosis of the RCA. Toxicology was negative as per the routine scope of analysis.

### 3.2 Case 2

A 14-year-old girl with a BMI of 35 ​kg/m^2^ was found to be unresponsive in the school playground in a remote location. She was noted to be ‘running and playing in normal fashion’ earlier. Her past history revealed her to have a cardiac murmur, epilepsy and polycystic ovarian syndrome (PCOS) for which she had undergone right oophorectomy. CPR was attempted. The cause of death was noted as acute cardiac failure due to Sudden Arrhythmic Death Syndrome in the context of obesity, non-alcoholic fatty liver disease, diabetes mellitus 2, respiratory tract infection, and physical exertion. At autopsy no clear macro- or microscopic cardiac pathology was found. There was extensive pulmonary edema, pericardial and pleural effusions, and fatty change of the liver. Toxicological analysis did not contribute to the formulation of a cause of death and no potential genetic cause of death was identified with cardiac genetic screening (cardiomyopathy and arrhythmic panels).

### 3.3 Case 3

A 28-year-old male was found to be unresponsive after collapsing in the toilet. This was soon after he had performed a large number of pushups. A past history of cigarette smoking, excessive alcohol use with alcohol dependence, alcoholic cardiomyopathy and liver disease was recorded. The cause of death was attributed to aortic arch thrombosis and alcoholic cardiomyopathy. Toxicology was negative as per the routine scope of analysis.

## 4 Discussion

In the present study, 59 cases of SCD were recorded with an annual incidence of 19.8 per 100,000 persons. The mean ​± ​SD age was 32.8 ​± ​6.14 years. 61 ​% (36/59) subjects were male. A total of 74.5 ​% (44/59) SCD occurred in remote areas with 61 ​% (36/59) occurring at home. The most common cause of SCD was CAD (61 ​%; 36/59) followed by RHD (8.5 ​%; 5/59) and SADS (7 ​%; 4/59). Comorbidities were common with a significant number having RHD (20.3 ​%; 12/59). Risk factors like cigarette smoking, alcohol abuse, obesity and substance abuse were noted in 30–37 ​% of cases.

Ha et al. looked at SCD related to physical exercise in subjects between 10 and 35 years over a period of 17 years. A total of 6 ​% of cases (n ​= ​110) were identified, with median age 27 years (IQR 21–32 years), and 92 ​% male gender. The common causes of SCD were CAD (37 ​%) followed by sudden arrhythmic death syndrome (SADS) (20 ​%), cardiomyopathy/cardiomegaly (15 ​%) and myocarditis (7 ​%). There were 10 cases in First Nations Australians with SCD due to CAD [12]. CAD was the leading cause of SCD in the present as well as the above study.

Young et al. presented a retrospective case series of 8 SCD due to IHD in young First Nations Australian sportsmen (mean age 29.4 years; range 21–36 years) in the Northern Territory between 1982 and 1996. It was estimated that the incidence of IHD related SCD among First Nations Australian football players in the Northern Territory was 19–24 per 100,000 player-years compared to 0.54 per 100,000 player-years among Australian Rules footballers of similar ages in Victoria. Based on this, the authors recommended screening of First Nations players younger than 40 years with risk factors for IHD [13].

Limited recent data is available on SCD in the Northern Territory. A retrospective review of coronial autopsies (179 adults) from the Northern Territory found that sudden death was 7.4 times higher and sudden death attributable to ischaemic heart disease (IHD) was 5.5 times higher in First Nations people compared to non-First Nations people [14]. In a study among children and young adults (1–35 years) in Australia and New Zealand by Bagnall et al., annual incidence of SCD was 1.3 cases per 100,000 persons, with CAD as cause SCD in 24 ​% of cases. In 31–35 years age group CAD was the cause in 45.6 ​% cases and 12.3 ​% in cases ≤30 years. The annual incidence of SCD in the present study was 15 times higher. Analysis to compare findings with Bagnall et al. study revealed CAD as the cause of death in 72.7 ​% cases (16/22) in 31–35 years age group and in 53.3 ​% of cases (8/15) ​≤ ​30 years. This suggests that CAD is common even in cases under 30 years of age in First Nations Australians in the Northern Territory [15].

Large numbers of First Nations Australian young people are undertaking sporting and physical activity on a regular or professional basis and should be encouraged to do so. This is a positive development overall, as regular exercise is associated with improved cardiovascular health along with benefits for other medical conditions, in a large majority of individuals. Wasfy et al. have cited epidemiological studies which consistently show benefit of moderate aerobic exercise in decreasing risk of CHD and death. However, for those with heart disease, avoidance of high intensity and prolonged exercise or competitive sports would be recommended. Awareness of the warning symptoms and signs would be important 255to stop exercise and seek medical help. In a small number of cases with certain cardiac conditions, exercise may be associated with increased risk of sudden death, referred to as ‘the exercise paradox’ [16].

The Australian government and other non-government agencies have prioritised placing an increased focus on supporting and developing First Nations Australian sport, physical activity, and health [[17], [18], [19]].

Among First Nations Australians, cardiovascular disease is one of the leading causes of death in the Northern Territory [20]. The prevalence of heart, stroke, and vascular disease was higher in First Nations population with First Nations patients requiring admission being younger and from more remote areas [21]. The average age of First Nations people affected by cardio-vascular diseases is 10–20 years younger than other Australians [6], with the Northern Territory having the highest total burden of disease as well as fatal burden [20].

The incidence of acute myocardial infarction in First Nations Australian population was three times that of other Australian population in the Northern Territory. In persons between 20 and 39 years of age this was nearly nine times, being more in men and residing in remote areas [21]. The age-standardised mortality rate was 67 ​% higher for First Nations Australians than non-first nations people, higher for men and remote residents [22]. This raises the issue of premature mortality due to CHD as a major sports cardiology issue in First Nations Australians.

In First Nations Australians, CHD accounted for 57 ​% of the CVD burden. In those aged 15–24 years, rheumatic heart disease was the leading cause of burden (25 ​%) while in those over 25 years, CHD accounted for more than half the burden [23]. In 2021, CHD was the leading cause of death in First Nations Australians with Northern Territory recording the highest mortality rate that was 2 times higher compared to non-First Nations Australians [20].

Overall, 2–3% of First Nations Australian people in the Northern Territory have known rheumatic heart disease (RHD). In the NT, the First Nations Australians make up only about 30 ​% of the population, but 90 ​% of all deaths from RHD occur among First Nations Australian population [24]. The First Nations age-standardized RHD prevalence is 61.4 times higher than non-First Nations Australians. Female RHD prevalence is double that in males. In a population-based study of 5 administrative regions in Australia, the highest prevalence (46.3 ​%) was estimated for the First Nations population of Northern Territory [25]. At the end of 2020, the NT had the highest prevalence rate for RHD with 78 ​% of them among First Nations Australians. The median age among First Nations Australians with RHD (34 years) was considerably younger (27 ​% under the age of 25 years) than for non-First Nations Australians (59 years) [23]. During the similar period, the highest incidence of RHD was among First Nations Australians in the Northern Territory. It was about twice as common in females and more than half of new cases were among First Nations Australians aged under 25 [26]. Consistent with higher prevalence of RHD in the Northern Territory, this was noted to be the second most common cause of SCD (5 cases) and an important co-existing cardiac condition in 7 cases, where there was another cause of death.

Other cardiac conditions like congestive heart failure, pulmonary hypertension and early repolarization patterns associated with risk of arrhythmia are also known to be more prevalent in First Nations Australians [[27], [28], [29]]. The rate of hospitalization among First Nations Australians with congenital heart disease including ventricular septal defect, atrial septal defect, patent ductus arteriosus, coarctation of aorta etc. is 1.2 times as high as the non- First Nations Australians [1].

A study of First Nations people aged 15–34 years found significant numbers with risk factors including elevated blood glucose (39 ​%), dyslipidaemia (73 ​%), smoking (63 ​%) and overweight/obesity (51 ​%) [30] In First Nations Australians, the major risk factors for CHD were dietary (45 ​%), high blood pressure (34 ​%), tobacco use (34 ​%) and being overweight/obese (33 ​%). Analysis of contribution of risk factors as proportion of total burden showed that tobacco use (11.9 ​%) was followed by being overweight/obese (9.7 ​%), diabetes (5.4 ​%), impaired kidney function (5 ​%), high blood pressure (4.3 ​%) and high cholesterol (3 ​%) [31]. In 30–37 ​% of patients in the present study, these risk factors were noted.

Diabetes mellitus and Chronic kidney disease (CKD) are known comorbid conditions with CVD and share a number of risk factors. The diabetes prevalence rate was 2.9 times higher among First Nations Australians compared to non–First Nations Australians [31]. In 2013 in the Northern Territory, First Nations youth represented 88 ​% of those diagnosed with diabetes [32]. In First Nations youth with type 2 diabetes, 59 ​% were noted to have hypertension, 24 ​% with dyslipidaemia and 61 ​% were obese [33]. Among First Nations Australian people, CKD was the 10th leading cause of total burden (2.5 ​% of all burden in 2018). This was higher for females (3.1 ​%) than males (2.3 ​%) [4]. In the present study, after cardiac comorbidities, diabetes mellitus (35.6 ​%) and renal disease (27.1 ​%) were the common co-morbidities.

SCD is rare but the leading medical cause of death in sports. It is catastrophic for the young athlete, their family and society as athletes are seen as physically fit individuals [34]. Some studies have indicated that involvement in sporting activities and training increases the risk of SCD 2.4 to 4.5 times when compared to non-athletes and recreational athletes [35,36]. Cardiovascular response to high level of physical training and risk of SCD is also impacted by ethnicity of the athlete. Specific cardiac and electrocardiographic abnormalities have been noted in Afro-Carribean [37,38], Asian [39], and West-Asian athlete population [40]. Pre-participation cardiovascular screening in Pacific Islands athletes reported cardio-vascular abnormality in 3.9 ​% of athletes with 0.8 ​% having risk of SCD. Cardiomyopathies, Wolff-Parkinson-White syndrome and valvular lesions of rheumatic origin were the main underlying conditions [41].

The ACSEP Position Statement on Pre-Participation Cardiac Evaluation in Young Athletes provides guidance on this topic in line with various international recommendations. ACSEP recommends evaluation of all young elite athletes for conditions linked to SCD using history, examination and resting 12 lead ECG. The age of commencement for pre-participation assessment for risk of SCD should be age 16 and continue bi-annually until the age of 25. It also acknowledges that the prevalence of IHD is higher in First Nations Australian people and that they may be at a higher risk of SCD [42].

Walsh and Kangaharan have suggested routine comprehensive cardiovascular health assessment to optimize cardiovascular care for First Nations Australians. This includes addressing risk factors, comorbidities and associated cardiac conditions [3]. Recent study suggests that there is greater potential for the use of health checks in improving identification and management of Aboriginal and Torres Strait Islander people at high risk of CVD, potentially preventing future CVD events [43].

The use of absolute risk approach to CVD risk assessment using multiple risk factors in First Nations Australians may underestimate the risk [44]. A recent consensus statement on cardiovascular disease risk assessment for First Nations Australian adults under the age of 35 years has pointed out the differences in recommendation from the commonly used guidelines in Australia. This is particularly in relation to age of commencement of absolute CVD risk assessment in First Nations Australians. Based on data that the proportion of First Nations Australian people aged 25–34 years at high risk of CVD is similar to that seen in non- First Nations Australian people aged 45–54 years, it is recommended that assessment and risk management should be carried out from an earlier age (18 years) [[44], [45], [46]]. In Aboriginal and Torres Strait Islander peoples without existing CVD: CVD risk factor screening should commence from the age of 18 years at the latest, including for blood glucose level or glycated haemoglobin, estimated glomerular filtration rate, serum lipids, urine albumin to creatinine ratio, and other risk factors such as blood pressure, history of familial hypercholesterolaemia, and smoking status [44].

Paige et al. have highlighted the lack of evidence on assessment and management of CVD risk in First Nations Australian people. The authors noted that CVD risk starts early and the routinely used guidelines may underestimate the CVD risk in this group [47].

Given the disproportionately high prevalence of RHD and burden of cardiovascular diseases and SCD in the Northern Territory First Nations Australians consideration needs to be given to a more comprehensive screening at an early age, with regular risk stratification. Echocardiography (ECHO) is essential and useful tool in the diagnosis and management of RHD (at any age) and coronary anatomy assessment should be considered for those at high risk. The new Australian CVD risk calculator endorsed in the Australian Guideline for assessing and managing cardiovascular disease risk is recommended for use to assess individual risk factors in First Nations Australians between 18 and 29 years of age [48].

This study had a few limitations. The review of Northern Territory SCD cases was of a coronial autopsy population and may not be representative of the entire population. While SCD cases are referred for coronial investigation in Australia, cases with a well-established known cardiac condition may not be reflected in this study. The SCD cases studied are likely to reflect the true number considering that during the study period 96.6 ​% of reported deaths in Northern Territory underwent an autopsy, the coroner completed investigations and issued a finding in 93.3 ​% of cases and the cause of death according to ICD-10 was recorded in 71.6 ​% of cases [49]. Another limitation is lack of information on socioeconomic status of the cases. From the study it is difficult to conclude that race is the predominant factor in the premature CAD, compared to socioeconomic disadvantage as data on socioeconomic status was not recorded. In future, The Aus CVD risk calculator that uses Socio-Economic Indexes for Areas (SEIFA) quintiles obtained from residential postcodes can be of help.

## 5 Conclusions

First Nations Australian population in Northern Territory has a disproportionately high burden of CVD resulting in a higher incidence of SCD at a very young age. Coronary artery disease was identified as the most common cause for SCD followed by RHD in this study. Unfortunately, current screening guidelines and risk stratification tools might underestimate the risk in this population. There is therefore a case for revising the current guidelines for screening to improve early detection of heart disease and risk factors in the First Nations Australians, at an earlier age of <16 years. There should be ongoing close clinical evaluation for early diagnosis, effective treatment to prevent SCD and guide decisions on their participation in sports. Further research and inclusion of data from Northern Territory in national clinical quality registries will provide ongoing insights into SCD in the First Nations Australians.

**Key Points**

• SCD is more common in First Nations Australians in the Northern Territory under 40 years of age, and coronary heart disease is the most common cause of SCD. RHD is more prevalent and the second leading cause.
• Current pre-participation screening is likely to underestimate the risk and causes of SCD in First Nation Australians population as this process relies on known health conditions.
• There is need for awareness of greater burden of SCD in younger First Nations Australian with First Nation specific guidelines for pre-participation screening at a younger age (<16 years).
• First Nations Australians in the Northern Territory should have access to cardiac health checks at a younger age with screening for RHD (using ECHO) and for CAD (coronary anatomy assessment).

## Declaration of generative AI and AI-assisted technologies in the writing process

No generative AI tool or AI-assisted technologies were used in the writing of this manuscript.

## Funding information

This research did not receive any specific external financial support. EP is supported by the Wilma Beswick Senior Research Fellowship at Melbourne University Sylvia & Charles Viertel Foundation, National Heart Foundation and Mamoma Foundation. EP has received speaker fees from Bristol Myers Squibb.

## Declaration of competing interest

The authors declare the following financial interests/personal relationships which may be considered as potential competing interests: Dr Elizabeth Paratz reports a relationship with The University of Melbourne that includes: funding grants. Dr Elizabeth Paratz reports a relationship with Bristol Myers Squibb Co that includes: speaking and lecture fees. If there are other authors, they declare that they have no known competing financial interests or personal relationships that could have appeared to influence the work reported in this paper.
